# Sex, Diastolic Blood Pressure, and Outcome after Thrombolysis for Ischemic Stroke

**DOI:** 10.1155/2014/747458

**Published:** 2014-09-15

**Authors:** David Nathanson, Cesare Patrone, Thomas Nyström, Mia von Euler

**Affiliations:** ^1^Karolinska Institutet, Department of Clinical Science and Education, Södersjukhuset, Stockholm, Sweden; ^2^Department of Internal Medicine, Sjukhusbacken 10 Södersjukhuset, 118 83 Stockholm, Sweden; ^3^Karolinska Institutet Stroke Research Network at Södersjukhuset, Stockholm, Sweden; ^4^Center for Gender Medicine, Karolinska Institutet, Stockholm, Sweden

## Abstract

*Background*. The goal of this study was to identify differences in risk factors and functional outcome between the two sexes in patients treated with thrombolysis for ischemic stroke. *Methods*. This cohort study audited data from patients treated with thrombolysis for ischemic stroke during a 3-year period at Södersjukhuset, Stockholm. *Results*. Of the 355 patients included in the study, 162 (45%) were women and 193 (54%) were men. Women were older with a median age of 76 years; median age for men was 69 years (*P* < 0.0001). Diastolic blood pressure was lower for women compared to men (*P* = 0.001). At admission fewer women had a favorable modified Rankin Scale score compared to men (93.8% versus 99%, *P* = 0.008). Three months after discharge functional status did not differ significantly between the two sexes. Diastolic blood pressure was associated to functional outcome only in men when sex specific odds ratios were calculated (OR, 5.7; 95% CI, 1.7–20). *Conclusion*. The study indicates that females appear to gain a relatively greater benefit from thrombolytic therapy than men due to a better functional recovery. A higher diastolic blood pressure increases the risk for a worse prospective functional status in men.

## 1. Introduction

Stroke is the primary cause of severe acquired disability in adults with 500,000 new cases each year in Europe [1, 2]. Administration of recombinant tissue plasminogen activator (rtPA), alteplase (Actilyse), within 4.5 h after onset of stroke, is an efficient treatment in patients where an intracerebral hemorrhage and other contraindications have been excluded [3–6]. Overall, women and men have a similar incidence for ischemic cerebrovascular disease but women are more frequently hit by stroke later in life than men [7]. Several epidemiological studies have shown that women having more severe stroke symptoms at admission, a worse prognosis, are less likely to return home and to live independently [8, 9] and have an overall worse outcome after ischemic stroke than men [10, 11]. However, some studies show a similar outcome for men and women after stroke [12, 13] and there is evidence that women treated with tPA benefit at least as much as men [14–16]. Very recently a study from the prospective multinational Safe Implementation of Treatments in Stroke International Stroke Thrombolysis Register (SITS-ISTR) suggested a possible larger beneficial effect of intravenous tPA in women compared with men [17]. Several recent studies have shown the same risk of bleeding and positive treatment effects in patients above 80 years old even though this age group has an overall higher mortality than younger patients [4, 18–20]. In the studies that form the scientific base for rtPA treatment for ischemic stroke, two thirds of the study populations were men [6, 21, 22]. A recent published systematic review of the literature did not find any major differences in the effect of thrombolysis between men and women although there was a trend towards a lower risk of symptomatic intracranial hemorrhage in women [22].

The primary aim of the present study was to investigate whether there are any differences, independent of age, in the basal risk factor profile between men and women with ischemic stroke treated with thrombolysis. Furthermore, the study seeks to assess any associations between these risk factors, sex, and outcome after thrombolysis (e.g., functional status and mortality after 3 months).

## 2. Methods

### 2.1. Participants

Since January 2008 all patients treated with rtPA for ischemic stroke at Södersjukhuset AB, a large teaching hospital in Stockholm, Sweden, have been consecutively registered in a local registry and followed for 3 months. The present study is a retrospective cohort study with prospectively collected data, including patients registered between January 2008 and December 2010 (censor date December 31, 2010) and discharged with a final diagnosis of transient ischemic attack or stroke. The study was approved by the regional ethical review board of Stockholm, EPN: 2012/626-31/4. Informed consent was obtained from the study participants.

### 2.2. Data Collection

Patient age, sex, door-to-needle time, vascular risk factors, blood pressure at admittance, CT scan at admittance and 24 h after thrombolysis, National Institute of Health Stroke Scale (NIHSS) score at admittance and at discharge, and an estimated Modified Rankin Scale (mRS) score before stroke were recorded. NIHSS and mRS scores at 3 months after stroke were obtained from the study participants at a face-to-face visit 3-month after thrombolysis. We evaluated the differences between men and women according to demographic data and pre-existing risk factors for stroke.

### 2.3. Clinical Assessment

Estimation of functional status was measured by mRS, which was assessed according to the Safe Implementation of Thrombolysis in Stroke-Monitoring Study (SITS-MOST) protocol [20, 23]. All mRS scoring was performed by one mRS- and NIHSS-certified study nurse. mRS score describing functional status for the patients before the stroke was collected within 48 hours after admission to hospital. A favourable mRS was defined as mRS ≤2.

Blood pressure was measured according to clinical routine in the left arm with the patient in a supine position.

SICH was assessed by the National Institute of Neurological Disorders and Stroke (NINDS) definition (any deterioration in NIHSS score or death within 7 days combined with intracerebral haemorrhage of any type on any posttreatment imaging after the start of thrombolysis).

### 2.4. Statistical Analyses

Means and standard deviations or medians and interquartile ranges were used to describe the characteristics of the study participants. Normal distribution of the variables was tested with Shapiro-Wilk's test. The independent samples *t*-test was used to compare means for normally distributed continuous variables. Skewed distributed variables were compared using Mann-Whitney test. Categorical variables were compared with *χ* ^2^ or Fisher's exact test. Logistic regression was used to investigate the associations between sex and hypertension and the outcomes: (a) 3-month favourable mRS and (b) 3-month mortality. First, univariable associations between outcomes, sex, and mortality were estimated. The results for diastolic blood pressure that differed between sexes at baseline are presented. The cut-off level (diastolic blood pressure <90 mmHg) was adopted from current European guidelines [24, 25].

Second, the identified associations were adjusted for known prognostic variables. To study if the association between sex and outcomes differed depending on the level of the diastolic blood pressure, the interaction between sex and blood pressure (sex∗diastolic blood pressure) was tested after associations between each interaction term (sex and diastolic blood pressure) and outcome had been investigated. Finally, we created a new categorized variable (sex∗diastolic blood pressure, cut-off level: 90 mmHg) from the variables in the interaction to study potential differences between males with high diastolic blood pressure versus females with low diastolic blood pressure. This new variable replaced the original variables in the final multivariable models.

Hosmer-Lemeshov goodness-of-fit test was used to examine whether the final multivariable models adequately fitted the data. Cooks distance was used to identify potential influential points in the multivariable analyses. To evaluate the influence of loss of data in the multivariable analyses, univariable analyses were performed with the population used in the multivariable analyses to determine whether these populations differed.

General linear models were used to investigate the influence of sex upon continuous variables (i.e., diastolic blood pressure, mean arterial blood pressure, and HDL-cholesterol).

All tests were 2-tailed; *P* value <0.05 was considered significant. All statistical analyses were performed with SPSS 20.0 software (Chicago, IL, USA).

## 3. Results

In total, there were 383 thrombolysis treatments. Patients who received more than one thrombolysis (*n* = 5) and patients who were discharged from hospital with main diagnoses other than ischemic stroke (*n* = 23) were excluded. Thus, 355 patients treated with rtPA for ischemic stroke were included in the analyses. In this cohort subtypes of ischemic stroke have not been listed. Of the 355 consecutive patients treated with rtPA and discharged from hospital with ischemic stroke as primary diagnosis, 162 (46%) were women and 193 (54%) were men. Women were older with a median age of 76 years while the median age for men was 69 years (*P* < 0.0001).

Antihypertensive drug treatment was given to 67% of the women prior the ischemic event (Table 1) whereas only 48% of men were treated for hypertension (*P* = 0.0003). Women were more often treated with diuretics (*P* = 0.0003) and *β*-blockers (*P* = 0.001) compared to men. Moreover a significantly larger proportion of the women were treated with acetyl salicylic acid (*P* = 0.009); 23.8% of the female subjects were treated with statins while 23.2% of men were on statins (*P* = 0.9).

Women had a significantly higher baseline NIHSS score, with a median of 7 (range = 1–27) compared to 5 in men (*P* = 0.006, range = 0–30) (Table 1). There were differences between female and male subjects according to pretreatment mRS with a poorer status in women (Table 2) while the functional mRS score 3 months after treatment was similar between men and women (Table 2). At admission, fewer women had a favourable 3-month mRS (93.8% versus 99%, *P* = 0.008) compared to men; 3 months after discharge the proportion having a favourable mRS did not differ significantly between men and women (Table 2).

In unadjusted analyses, women had a higher prevalence for atrial fibrillation (OR, 2.1; 95% CI, 1.3–3.6, *P* = 0.005) and hypertension (OR, 1.7; 95% CI, 1.1–2.6, *P* = 0.02) but when adjusted for age these differences became insignificant (OR, 1.6; 95% CI, 0.96–2.9, *P* = 0.08 and OR, 1.3; 95% CI, 0.8–2.0, *P* = 0.25, resp.). Baseline diastolic blood pressure was lower for women than for men (Table 1). This difference remained significant when adjusted for age and BMI in general linear models (adjusted mean difference, 4.9 mmHg; *P* = 0.001). HDL-cholesterol levels were higher for female subjects both in unadjusted analyses (Table 1) and after adjustment for age and BMI (adjusted mean difference, 0.21 mmol/L; *P* = 0.0001). Among the 19 patients (5.4%) who died before discharge from hospital, 11 (5.7%) were men and 8 (4.9%) were women. The 3-month mortality for the entire cohort was 10.7% (*n* = 38) and was 7.7% (*n* = 15) for men and 14.2% (*n* = 23) for women. There was no significant difference between the two sexes in 3-month (*P* = 0.08) or in-hospital mortality (*P* = 0.8). Neither was there any difference between men and women in incidence of SICH (NINDS), with an incidence of 4.7% (*n* = 9) and 5.6% (*n* = 9) in men and women, respectively (*P* = 0.8).

Univariable analyses showed no associations between sex or blood pressure and the outcomes (mRS and mortality) (Table 3). After adjustment for relevant risk factors a significant association between diastolic blood pressure and favourable mRS was revealed (Table 3(a)). We did not find any association between systolic blood pressure and outcome (data not shown). When sex specific odds ratios were calculated diastolic blood pressure <90 mmHg predicted favourable functional outcome in men (OR, 5.7; 95% CI, 1.7–20) but not in women (OR, 1.5; 95% CI, 0.3–8.1).

The one-at-a-time interaction analyses test (sex∗diastolic blood pressure) revealed a reduced chance for a favourable functional outcome among men with high diastolic blood pressure (Table 3(a)).

No significant associations could be found in similar analyses when the outcome was 3-month mortality (Table 3(b)). Finally, there were no significant interactions between HDL-cholesterol, sex, and outcome (data not shown).

## 4. Discussion

In the present study, we found that the outcomes after treatment with rtPA for ischemic stroke are similar between women and men despite women being older, having worse pretreatment functional status (mRS and NIHSS) and having more cardiovascular risk factors compared to men. Further, differences in prevalence for atrial fibrillation and hypertension mainly depend on age as these associations become insignificant when adjusted for age. This is in accordance with earlier studies showing women to have a greater benefit from thrombolytic therapy for ischemic stroke compared to men [14, 15, 17, 26]. The poorer outcome after stroke in women has commonly been explained by women being older at stroke onset, having a lower function level before stroke onset and having larger strokes than men [8, 9]. The possibility of erasing this difference may indicate that the most important factor for the poorer outcome in women is largely due to more severe strokes and thrombolysis may counteract this. The finding from several studies that women receive less aggressive emergency treatment than men is thus a concern [27, 28]. Particularly as the longevity advantage of women allows more of them to reach the age of greatest stroke risk [29]. The therapeutic response to rtPA might depend on the cause of ischemia [16]. Furthermore, successful recanalization is more probable if the occlusion is caused by a fibrin-rich cardioembolic embolus rather than platelet-rich thromboses from atherosclerotic lesions, due to the high affinity of rtPA for fibrin [16, 30]. The significantly higher prevalence of cardioembolic causes of stroke (i.e., atrial fibrillation) found in this and previous studies [12, 13] can explain the relatively greater improvement in functional status in women. Another potential factor that can protect against SICH, thus allowing a similar outcome between men and women after rtPA, is the finding of a lower diastolic blood pressure of women compared to men. In multivariable analyses we found a significant sex-by-diastolic blood pressure interaction for 3-month favourable mRS where a diastolic blood pressure <90 mmHg in female subjects rendered a 7.9 times higher chance of a favourable mRS 3 months after the ischemic event compared to men with a diastolic blood pressure >90 mmHg. In our study high diastolic blood pressure predicted lower chance for favourable functional outcome in men but not in women. There are several studies showing that high systolic blood pressure is an independent risk factor for all causes of mortality in ischemic stroke [31, 32]. Although there are few studies indicating that high diastolic blood pressure is an independent risk-factor for functional outcome. In a prospective study on patients with acute ischemic stroke, Manabe et al. showed that high blood pressure in the acute phase of stroke was associated with a higher mRS score [33]. In their study, there was a strong association between high blood pressure and fatal brain edema and early recurrent stroke. Also, recently demonstrated by Caso et al., high diastolic blood pressure on admission in acute stroke patients correlated with in-hospital mortality while systolic blood pressure did not. In this study the risk of death increased by 5% for each 1 mm increase of diastolic blood pressure [34]. A high diastolic blood pressure might augment edema formation as the major cause of cerebral edema is increased capillary pressure which in turn is dependent of mean arterial blood pressure [34].

Brain edema or recurrent stroke has not been assessed in the current study, but it is likely that these complications can be potential explanations for the association between higher diastolic blood pressure and a worse functional outcome.

We did not find any significant associations when the outcome was 3-month mortality, although the sample might have been too small and with too few events to reveal any associations between sex, blood pressure, and mortality.

In the present study, a larger proportion of the women were treated with antihypertensive agents, especially diuretics, prior to the stroke, which might have caused the lower diastolic blood pressure in women compared to men. Nevertheless, the associations between sex, blood pressure, and functional status remained significant when adjusted for antihypertensive treatment and acetyl salicylic acid treatment.

Limitations of the present study are the relatively small sample size and the observational study design that carries a risk of residual and unmeasured confounding such as general health prestroke and other socioeconomic factors [35]. Moreover, the population studied is a cohort with mild stroke symptoms which further limits the generalizability of the findings. However, in the Swedish national quality register (to which all hospitals treating stroke report) NIHSS was 7 in 2013, which was the same as in the women, but slightly higher than the NIHSS found in men in our cohort [36].

The strength of the study is the internal validity as the data collection and functional assessment were made by one mRS- and NIHSS-certified study nurse.

## 5. Conclusion

In conclusion, this cohort study demonstrates differences in risk factor profiles between men and women treated with thrombolytic therapy for ischemic stroke.

The study indicates that females appear to gain a relatively greater benefit from thrombolytic therapy than men due to a better functional recovery. A higher diastolic blood pressure increases the risk for a worse prospective functional status in men but seems to be of less significance in women.

## Figures and Tables

**Table 1:** Baseline characteristics.

	Total (*n* = 355)	Men (*n* = 193)	Women (*n* = 162)	*P* value
Age	71 (63–71)	69 (61–76)	76 (67–84)	<0.0001
Medical history				
Atrial fibrillation	73 (20.6)	29 (15)	44 (27.2)	0.005
Diabetes	51 (14.4)	23 (11.9)	28 (17.3)	0.2
Smoke currently	77 (21.7)	43 (23.4)	34 (22.5)	0.89
BMI	26 ± 4.4	26 ± 4.0	26 ± 13.0	0.9
Hypertension	168 (47.3)	80 (41.5)	88 (54.3)	0.02
Clinical assessment				
Baseline NIHSS score	5 (3–11)	5 (3–9)	7 (3–14)	0.006
Systolic BP, mmHg	152 ± 20	152 ± 20	152 ± 20	0.9
Diastolic BP, mmHg	81 ± 13	84 ± 13	78 ± 12	<0.0001
Diastolic BP >90 mmHg	78 (22)	55 (28.5)	23 (14.2)	0.001
MAP, mmHg	104 ± 13	107 ± 13	102 ± 12	0.002
Laboratory findings				
Glucose at admission, mmol/L	7.0 ± 2.4	7.1 ± 2.7	6.8 ± 2.7	0.18
Triglycerides, mmol/L	1.4 ± 0.9	1.4 ± 0.8	1.4 ± 1.0	0.8
LDL-cholesterol, mmol/L	3.1 ± 1.0	3.1 ± 1.0	3.0 ± 0.9	0.4
HDL-cholesterol, mmol/L	1.4 ± 0.4	1.3 ± 0.4	1.5 ± 0.5	0.0001
CRP	6.1 ± 14	5.7 ± 13	6.6 ± 16	0.6
Creatinine clearance, mL/min	76 ± 32	86 ± 32	65 ± 29	<0.0001
Treatment				
Statins	82 (23.1)	44 (23.2)	38 (23.8)	0.9
ASA	146 (41.1)	67 (35.3)	79 (49.1)	0.009
Antihypertensive treatment	196 (55.2)	89 (47.6)	107 (66.9)	0.0003
B-blocker	135 (38.0)	57 (30.5)	78 (48.8)	0.001
Diuretics	61 (17.2)	20 (10.8)	41 (25.6)	0.0003
ACE inhibitors	62 (17.5)	37 (19.9)	25 (15.6)	0.3
Angiotensin receptor blockers	30 (8.5)	13 (7.0)	17 (10.6)	0.2
Calcium channel blockers	56 (15.8)	28 (15.1)	28 (17.5)	0.5
Door-needle, minutes	60 (50–77)	60 (51–78)	59 (49–77)	0.8

Abbreviations: BMI: body mass index; BP: blood pressure; NIHSS: National Institute of Health Stroke Scale; IGT: impaired glucose tolerance; MAP: mean arterial pressure; OGTT: oral glucose tolerance test; OGTT-120 min, 120 minutes post glucose challenge during oral glucose tolerance test; mRS: modified rankin scale; CRP: c reactive protein; LDL: low density lipoprotein; HDL: high density lipoprotein; ASA: acetyl salicylic acid.

Data are described as *n* (%), means ± SD or median with interquartile range in parentheses. Creatinin-clearance is calculated from the Cockroft-Gault formula.

**Table 2:** Modified Rankin Scale score before ischemic event and 3 months after rtPA.

	Men (*n* = 193)	Women (*n* = 162)	*P* value
BASELINE mRS SCORE			
0 (no symptoms at all)	158 (82.7)	107 (66.5)	0.0004
1 (no significant disability despite symptoms)	20 (10.5)	35 (21.7)	0.004
2 (slight disability)	11 (5.8)	9 (5.6)	0.9
3 (moderate disability)	1 (0.5)	8 (5.0)	0.008
4 (moderate severe disability)	1 (0.5)	2 (1.2)	0.5
5 (severe disability)	0 (0)	0 (0)	NS
Favourable mRS score	189 (99.0)	151 (93.8)	0.008
3-MONTH mRS SCORE			
0 (no symptoms at all)	56 (29.0)	42 (25.9)	0.2
1 (no significant disability despite symptoms)	38 (23.5)	33 (22.1)	0.8
2 (slight disability)	20 (12.3)	24 (16.1)	0.3
3 (moderate disability)	14 (7.3)	10 (6.7)	0.5
4 (moderate severe disability)	14 (8.6)	13 (8.7)	1.0
5 (severe disability)	5 (2.6)	4 (2.5)	0.8
6 (dead)	15 (9.3)	23 (15.4)	0.1
Favourable mRS score	114 (70.4)	99 (66.4)	0.5

Abbreviations: mRS: modified rankin scale.

Data are described as *n* (%); *P* values were calculated and proportions were compared using the *χ* ^2^ test.

**(a):** 

	*n* (% favourable mRS)	Univariable			Multivariable^a^			Multivariable^b^			Multivariable^c^		
		OR	95% CI	*P*	OR	95% CI	*P*	OR	95% CI	*P*	OR	95% CI	*P*
Sex^1^													
Men	162 (70.4)	Reference											
Women	149 (66.4)	0.8	0.5–1.3	0.5	0.9	0.5–1.8	0.9	1.7	0.7–3.9	0.2			
DBP^2^													
>90 mmHg	68 (63.2)	Reference											
<90 mmHg	240 (70.0)	1.4	0.7–2.4	0.3				3.6	1.5–8.9	0.005			
DBP males													
>90 mmHg	46 (60.9)	Reference											
<90 mmHg	115 (74.8)	1.9	0.9–3.9	0.08							5.7	1.7–20	0.006
DBP females													
>90 mmHg	22 (68.2)	Reference											
<90 mmHg	125 (65.6)	0.9	0.4–2.3	0.8							1.5	0.3–8.1	0.6
Interactions sex and DBP													
Male >90 mmHg	46 (60.9)										Reference		
Female >90 mmHg	22 (68.2)										5.3	0.9–32.0	0.07
Male <90 mmHg	115 (74.8)										5.9	1.9–18.7	0.001
Female <90 mmHg	125 (65.6)										7.9	2.4–26.0	0.001

Abbreviations: DBP: diastolic blood pressure; mRS: modified rankin scale; 3-month favorable mRS: 3 month modified rankin scale ≤2.

Model 1^a^: associations adjusted for hypertension, cholesterol and HDL-cholesterol and diastolic blood pressure. Model 1^b^: associations adjusted for age, baseline NIHSS, favourable mRS before treatment, hypertension, cholesterol and HDL-cholesterol and diastolic blood pressure. Model 2^b^: associations adjusted for age, baseline NIHSS, favourable mRS before treatment, cholesterol and HDL-cholesterol, sex, ASA and antihypertensive treatment. Model^c^: associations adjusted for age, baseline NIHSS, favourable mRS before treatment, hypertension, cholesterol, HDL-cholesterol, ASA and antihypertensive treatment. In the final multivariable analyses 247 patients were included.

**(b):** 

	*n* (% mortality)	Univariable			Multivariable^a^			Multivariable^b^		
		OR	95% CI	*P*	OR	95% CI	*P*	OR	95% CI	*P*
Sex^1^										
Women	159 (14.5)	Reference								
Men	185 (8.1)	0.5	0.3–1.0	0.06	2.1	0.5–9.3	0.3			
DBP^2^										
>90 mmHg	77 (9.1)	Reference								
<90 mmHg	264 (9.1)	1.3	0.6–3.1	0.5				0.7	0.2–2.9	0.6
DBP males										
>90 mmHg	54 (9.3)	Reference								
<90 mmHg	130 (7.7)	0.8	0.3–2.5	0.7				0.6	0.1–5.8	0.5
DBP females										
>90 mmHg		Reference								
<90 mmHg		1.9	0.4–8.9	0.4				4.3	0.1–143	0.4
Interactions sex and DBP^3^										
Male >90 mmHg	54 (9.3)							Reference		
Female >90 mmHg	23 (8.7)							0.05	0.01–2.0	0.4
Male <90 mmHg	130 (7.7)							0.3	0.03–2.4	0.2
Female <90 mmHg	134 (15.7)							0.1	0.02–1.7	0.1

Abbreviations: DBP: diastolic blood pressure; mRS: modified rankin scale; 3-month favorable mRS: 3 month modified rankin scale ≤2. Model 1^a^: associations adjusted for age, baseline NIHSS, favourable mRS before treatment, hypertension, cholesterol and HDL-cholesterol and diastolic blood pressure. Model 2^b^: associations adjusted for age, sex, baseline NIHSS, favourable mRS before treatment, cholesterol, HDL-cholesterol, acetyl salicylic acid and antihypertensive treatment. Model 3^b^: associations adjusted for age, baseline NIHSS, favourable mRS before treatment, hypertension, cholesterol, HDL-cholesterol, acetyl salicylic acid, hypertension and antihypertensive treatment. In the final multivariable analyses 247 patients were included.
